# Spontaneous Magnetization Induced by Antiferromagnetic Toroidal Ordering

**DOI:** 10.3390/nano14211729

**Published:** 2024-10-29

**Authors:** Satoru Hayami

**Affiliations:** Graduate School of Science, Hokkaido University, Sapporo 060-0810, Japan; hayami@phys.sci.hokudai.ac.jp

**Keywords:** multipole, magnetic toroidal dipole, zigzag chain, antiferromagnets, magnetization, antisymmetric spin-orbit interaction

## Abstract

The magnetic toroidal dipole moment, which is induced by a vortex-type spin texture, manifests itself in parity-breaking physical phenomena, such as a linear magnetoelectric effect and nonreciprocal transport. We elucidate that a staggered alignment of the magnetic toroidal dipole can give rise to spontaneous magnetization even under antiferromagnetic structures. We demonstrate the emergence of uniform magnetization by considering the collinear antiferromagnetic structure with the staggered magnetic toroidal dipole moment on a bilayer zigzag chain. Based on the model calculations, we show that the interplay between the collinear antiferromagnetic mean field and relativistic spin-orbit coupling plays an important role in inducing the magnetization.

## 1. Introduction

**Figure 1:**
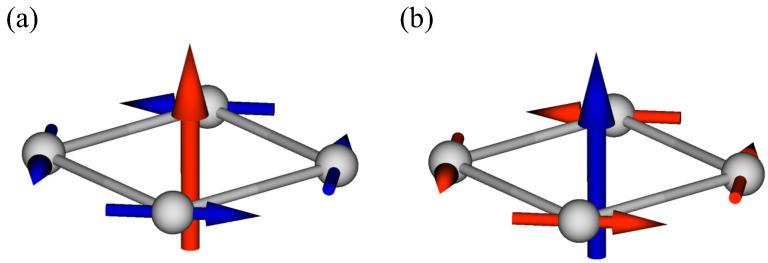
Schematic pictures of (**a**) the magnetic toroidal dipole (red arrow) consisting of magnetic dipoles (blue arrows) and (**b**) the magnetic dipole consisting of magnetic toroidal dipoles.

A magnetic toroidal dipole (MTD) moment has long been studied in condensed matter physics [1,2,3,4,5,6,7,8,9,10,11,12], since it becomes a microscopic origin of parity-violating physical phenomena, such as the linear magnetoelectric effect [13,14,15,16,17], asymmetric magnon excitations [18,19,20,21], nonlinear nonreciprocal transport [22,23,24,25,26,27], and nonlinear spin Hall effect [28,29]. The microscopic expression of the MTD is given by

(1) $$\begin{array}{c} T=\sum_{i}r_{i}\times S_{i}, \end{array}$$

where ***r***_*i*_ and ***S***_*i*_ represent the position vector and localized spins at site *i*, respectively; we omit the coefficient for notational simplicity. One of the typical magnetic structures to possess nonzero ***T*** is a vortex-type spin structure, as schematically shown in Figure 1a. Such a vortex-type spin texture has been found in UNi_4_B [30,31,32,33], where the MTD-related physical phenomena have been observed in experiments [34,35]. Since the MTD is not only induced in noncollinear antiferromagnetic (AFM) structures but also in collinear ones, there are a variety of magnetic materials, such as Cr_2_O_3_ [13,36,37], GaFeO_3_ [38,39,40,41], LiCoPO_4_ [42,43,44,45], Ba_2_CoGe_2_O_7_ [46,47,48,49], LiFeSi_2_O_6_ [50,51,52], CuMnAs [53,54,55,56,57], BaCoSiO_4_ [58,59], and PbMn_2_Ni_6_Te_3_O_18_ [60]. Recently, it was recognized that the materials with the zigzag chain, such as NdRu_2_Al_10_ and TbRu_2_Al_10_, are typical materials to possess the MTD in collinear AFM structures, since the simple staggered collinear AFM orderings naturally lead to the MTD moment [16,61].

Meanwhile, one notices that the magnetization (magnetic dipole) is related to the vortex-type configuration of the MTD by replacing ***T*** and ***S***_*i*_ in Equation (1) with each other. In other words, one can engineer spontaneous magnetization by considering the spatial distribution of the MTD from the symmetry viewpoint. With this close relationship between the magnetic dipole and MTD in mind, we propose a way how to generate spontaneous magnetization in the AFM structure accompanying the antiferro MTD moment. By considering a bilayer zigzag-chain system as an example, we demonstrate that the AFM ordering with the staggered MTD moment can naturally give rise to a uniform magnetization perpendicular to both AFM and MTD moments by performing the symmetry and microscopic model analyses. We show that the relativistic spin-orbit coupling is essential to induce the uniform magnetization. Our results provide an intuitive interpretation of why some antiferromagnetic ordered states with a negligibly small uniform magnetization exhibit the anomalous Hall effect.

The rest of this paper is organized as follows. In Section 2, we introduce the bilayer zigzag chain as a prototype to possess uniform magnetization by the staggered MTD moment. First, from the symmetry viewpoint, we show the relationship between the magnetic dipole and MTD under the magnetic point group. Then, we construct a minimal tight-binding model to capture the essence of spontaneous magnetization in AFM with the staggered MTD. In Section 3, we show that the electronic band structure under the staggered MTD ordering exhibits the Zeeman splitting. We also show that the spin-orbit coupling is an important parameter at the microscopic level. Section 4 is devoted to the conclusion of the present paper.

## 2. Setup

### 2.1. Symmetry Analysis

**Figure 2:**
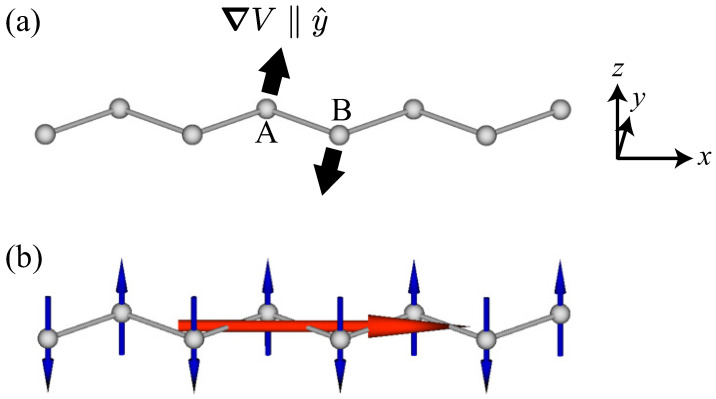
(**a**) Schematic picture of the single zigzag-chain structure consisting of two sublattices, A and B. In each sublattice, the opposite potential gradient occurs in the *y* direction. ∇V represents the potential gradient arising from the lack of local inversion symmetry at the lattice site. (**b**) The staggered antiferromagnetic ordering, which induces the MTD moment along the *x* direction; the blue and red arrows stand for the spin and MTD, respectively. The picture is drawn by MultiPie [79].

First, we consider the symmetry correspondence between the MTD moment and antiferromagnetic structures. Let us consider the single zigzag chain along the *x* direction, as shown in Figure 2a. The zigzag-chain system consists of two sublattices, which are denoted by A and B in Figure 2a. Although there is a global inversion center at the bond center between A and B sublattices, the local inversion symmetry is absent at each lattice site, which leads to the local potential gradient along the *y* direction, as shown in Figure 2a. It is noted that the direction of the potential gradient for the A sublattice is opposite to that for the B sublattice owing to the global inversion symmetry. In such a situation, the staggered AFM ordering with the *z*-spin polarization can lead to the MTD moment along the *x* direction [16,61,62], as schematically shown in Figure 2b. This is understood from the fact that the potential gradient has the same symmetry as the position vector, i.e., $\sum_{i}r_{i}\times S_{i}\propto \sum_{i}\nabla V_{i}\times S_{i}$. When the potential gradient and the spin moment are along the *y* and *z* directions, respectively, the resultant MTD moment is along the *x* direction. A similar situation also occurs in other locally noncentrosymmetric lattice structures, such as the honeycomb structure [21,63,64,65,66,67,68], diamond structure [69,70,71,72,73,74], and other structures [75,76,77].

From the symmetry viewpoint, the zigzag-chain system belongs to the orthorhombic system under the $D_{2h}$ symmetry. When the staggered AFM ordering with the *z*-spin polarization occurs, the irreducible representation $B_{3u}^{-}$ belongs to the totally symmetric irreducible representation, where the superscript of the irreducible representation represents the time-reversal parity. Since the *x*-component MTD moment also belongs to the $B_{3u}^{-}$ representation [78], one finds that the staggered AFM ordering accompanies the uniform MTD moment along the *x* direction. It is noted that different types of multipoles are induced when the direction of the AFM moment changes; the magnetic monopole $M_{0}$ (the *z*-component MTD) is induced when the AFM moment lies in the *y* (*x*) direction. We show the correspondence between the multipoles and staggered AFM structures in Table 1.

**Figure 3:**
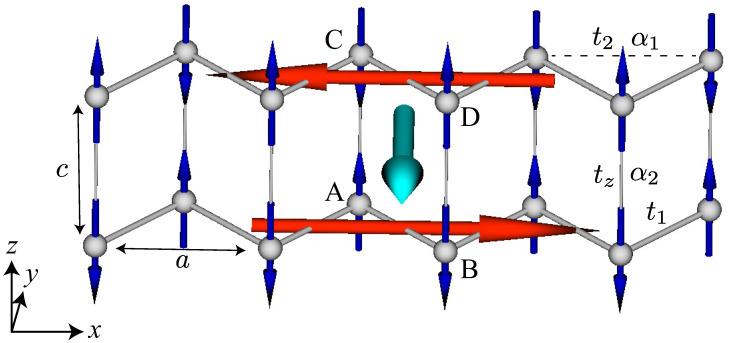
Antiferromagnetic structure in the bilayer zigzag-chain structure consisting of four sublattices A, B, C, and D. In each zigzag chain, the magnetic toroidal dipole moment denoted by the red arrows along the *x* direction is induced in the staggered alignment of the magnetic dipole (spin) moment denoted by the blue arrows along the *z* direction. The staggered alignment of the magnetic toroidal dipole moment leads to the uniform magnetization denoted by the cyan arrows along the *y* direction. The picture is drawn by MultiPie [79].

Next, we consider the AFM structure on the bilayer zigzag chain with four sublattices; the zigzag chain is stacked along the *z* direction with the same xy position, as shown in Figure 3; the crystal symmetry is the same as that in the single zigzag-chain system. When the AFM structure within the single zigzag chain is stacked ferromagnetically, the induced multipole moments are the same as those in the single zigzag-chain system, as shown in Table 1. On the other hand, in the case of the AFM stacking shown in Figure 3, the induced multipole moments are different due to the recovery of the spatial inversion symmetry; the inversion center is located at the nearest-neighbor bonds along the *z* direction. Indeed, the direction of the MTD induced by the staggered AFM structure is opposite for different zigzag chains so that its net component is canceled out. The irreducible representation of the *z*-polarized AFM structure corresponds to $B_{2g}^{-}$, as shown in Table 1. Since the magnetic dipole $M_{y}$ also belongs to $B_{2g}^{-}$, the AFM structure in Figure 3 is expected to have a uniform magnetization along the *y* direction. The emergence of uniform magnetization is also understood from the spatial alignment of the MTD; the staggered MTD induces uniform magnetization along the perpendicular direction according to the relation $M\propto \sum_{i}r_{i}\times T_{i}$, as discussed in the introduction. Similarly, the AFM stacking with the *y*-spin polarization induces the net magnetization along the *z* direction. Meanwhile, the AFM stacking with the *x*-spin polarization induces no net magnetization; instead, the magnetic toroidal monopole $T_{0}$, which is characterized by a source of the MTD, i.e., $T_{0}\propto \sum_{i}r_{i}\cdot T_{i}$, is induced [80].

### 2.2. Model

To investigate the behavior of the uniform magnetization under the staggered MTD on the bilayer zigzag chain consisting of four sublattices A, B, C, and D, we consider the fundamental tight-binding model, whose Hamiltonian is given by

(2) $$\begin{array}{cc} H= & -t_{1}\sum_{\langle i,j\rangle \sigma }c_{i\sigma }^{†}c_{j\sigma }-t_{2}\sum_{\langle \langle i,j\rangle \rangle \sigma }c_{i\sigma }^{†}c_{j\sigma }-t_{z}\sum_{{\langle i,j\rangle }_{z}\sigma }c_{i\sigma }^{†}c_{j\sigma }+i\alpha_{1}\sum_{\langle \langle i,j\rangle \rangle \sigma \sigma^{\prime }}p\left(i\right)\sigma_{z}c_{i\sigma }^{†}c_{j\sigma^{\prime }}\\ \; & +i\alpha_{2}\sum_{{\langle i,j\rangle }_{z}\sigma \sigma^{\prime }}p\left(i\right)\sigma_{x}c_{i\sigma }^{†}c_{j\sigma^{\prime }}-h^{\mathrm{FMT}}\sum_{i}T_{z}^{\mathrm{FMT}}c_{i\sigma }^{†}c_{i\sigma }-h^{\mathrm{AFMT}}\sum_{i}T_{z}^{\mathrm{AFMT}}c_{i\sigma }^{†}c_{i\sigma }, \end{array}$$

where $c_{i\sigma }^{†}$ ($c_{i\sigma }$) is the creation (annihilation) operator of electrons at site *i* and spin σ=↑,↓. The first, second, and third terms represent the hopping of electrons with the amplitudes of $t_{1}$, $t_{2}$, and $t_{z}$, respectively; 〈⋯〉, 〈〈⋯〉〉, and ${\langle \cdots \rangle }_{z}$ stand for the nearest-neighbor pair along the *x* direction, the next-nearest-neighbor pair along the *x* direction, and the nearest-neighbor pair along the *z* direction, respectively. The fourth and fifth terms represent the spin-dependent imaginary hopping that arises from the relativistic spin-orbit coupling; p(i) is +1 (−1) when the site *i* indicates the sublattices A and C (B and D) and $\sigma_{\mu }$ represents the μ=x,y,z component of the 2×2 Pauli matrix. These terms correspond to the sublattice-dependent antisymmetric spin-orbit interaction (ASOI) characteristic of the locally noncentrosymmetric lattice systems. Indeed, the Fourier transform of these terms leads to the form of ASOI as g(k)·σ, where g(k) is the so-called *g*-vector and it is given by $g\left(k\right)=[0,0,\pm \alpha_{1}\sin\left(k_{x}a\right)]$ along the $k_{x}\equiv k$ direction [+ (−) is for the sublattice A (B)]. It is noted that the opposite sign of g(k) is owing to the presence of the global inversion symmetry of the lattice structure. In the first to fifth terms in the Hamiltonian, the contribution from the Hermite conjugation is implicitly taken into account. The 4×4 matrix elements of the hopping Hamiltonian in the first to third terms of Equation (2), $H^{4\times 4}$, is given in momentum space as follows:

(3) $$\begin{array}{c} H^{4\times 4}=\left(\begin{array}{cccc} -2t_{2}\cos k & -2t_{1}\cos\frac{k}{2} & -t_{z} & 0\\ -2t_{1}\cos\frac{k}{2} & -2t_{2}\cos k & 0 & -t_{z}\\ -t_{z} & 0 & -2t_{2}\cos k & -2t_{1}\cos\frac{k}{2}\\ 0 & -t_{z} & -2t_{1}\cos\frac{k}{2} & -2t_{2}\cos k \end{array}\right), \end{array}$$

where the basis is given by $\left\{c_{A\sigma },c_{B\sigma },c_{C\sigma },c_{D\sigma }\right\}$. Similarly, the 8×8 matrix elements of the ASOI Hamiltonian in the fourth and fifth terms of Equation (2), $H^{8\times 8}$, is given in momentum space as follows:

(4) $$\begin{array}{c} H^{8\times 8}=\left(\begin{array}{cccccccc} 2\alpha_{1}s_{k} & 0 & 0 & 0 & 0 & 0 & -i\alpha_{2} & 0\\ 0 & -2\alpha_{1}s_{k} & 0 & 0 & 0 & 0 & 0 & i\alpha_{2}\\ 0 & 0 & 2\alpha_{1}s_{k} & 0 & i\alpha_{2} & 0 & 0 & 0\\ 0 & 0 & 0 & -2\alpha_{1}s_{k} & 0 & -i\alpha_{2} & 0 & 0\\ 0 & 0 & -i\alpha_{2} & 0 & -2\alpha_{1}s_{k} & 0 & 0 & 0\\ 0 & 0 & 0 & i\alpha_{2} & 0 & 2\alpha_{1}s_{k} & 0 & 0\\ i\alpha_{2} & 0 & 0 & 0 & 0 & 0 & -2\alpha_{1}s_{k} & 0\\ 0 & -i\alpha_{2} & 0 & 0 & 0 & 0 & 0 & 2\alpha_{1}s_{k} \end{array}\right), \end{array}$$

where $s_{k}=\sin k$ and the basis is given by $\left\{c_{A↑},c_{B↑},c_{C↑},c_{D↑},c_{A↓},c_{B↓},c_{C↓},c_{D↓}\right\}$.

The sixth and seventh terms represent the mean fields corresponding to the uniform and staggered MTD orderings with the *z*-spin polarization, respectively. In the case of the uniform MTD ordering, the 8×8 matrix for the $T_{z}^{\mathrm{FMT}}$ is given by

(5) $$\begin{array}{c} T_{z}^{\mathrm{FMT}}=\left(\begin{array}{cccc} \sigma & 0 & 0 & 0\\ 0 & -\sigma & 0 & 0\\ 0 & 0 & \sigma & 0\\ 0 & 0 & 0 & -\sigma \end{array}\right), \end{array}$$

where the basis is given by $\left\{c_{A\sigma },c_{B\sigma },c_{C\sigma },c_{D\sigma }\right\}$ and σ=+1(−1) for ↑ (↓). This mean field corresponds to the “FMT" in Table 1. Similarly, the 8×8 matrix for the $T_{z}^{\mathrm{AFMT}}$ to induce the staggered MTD ordering is given by

(6) $$\begin{array}{c} T_{z}^{\mathrm{AFMT}}=\left(\begin{array}{cccc} \sigma & 0 & 0 & 0\\ 0 & -\sigma & 0 & 0\\ 0 & 0 & -\sigma & 0\\ 0 & 0 & 0 & \sigma \end{array}\right). \end{array}$$

This mean field corresponds to the “AFMT” in Table 1. The schematic AFM structure to have the staggered MTD is shown in Figure 3. Since both the effects of the ASOI and the collinear AFM mean field, which are necessary for inducing the uniform magnetization as detailed below, are included in the model Hamiltonian in Equation (2), the present model corresponds to a minimal model to clarify the relationship between the staggered MTD and the uniform magnetization, whose results in the subsequent section can be straightforwardly applied for different models by changing the model parameters appropriately.

## 3. Results

### 3.1. Electronic Band Structure

**Figure 4:**
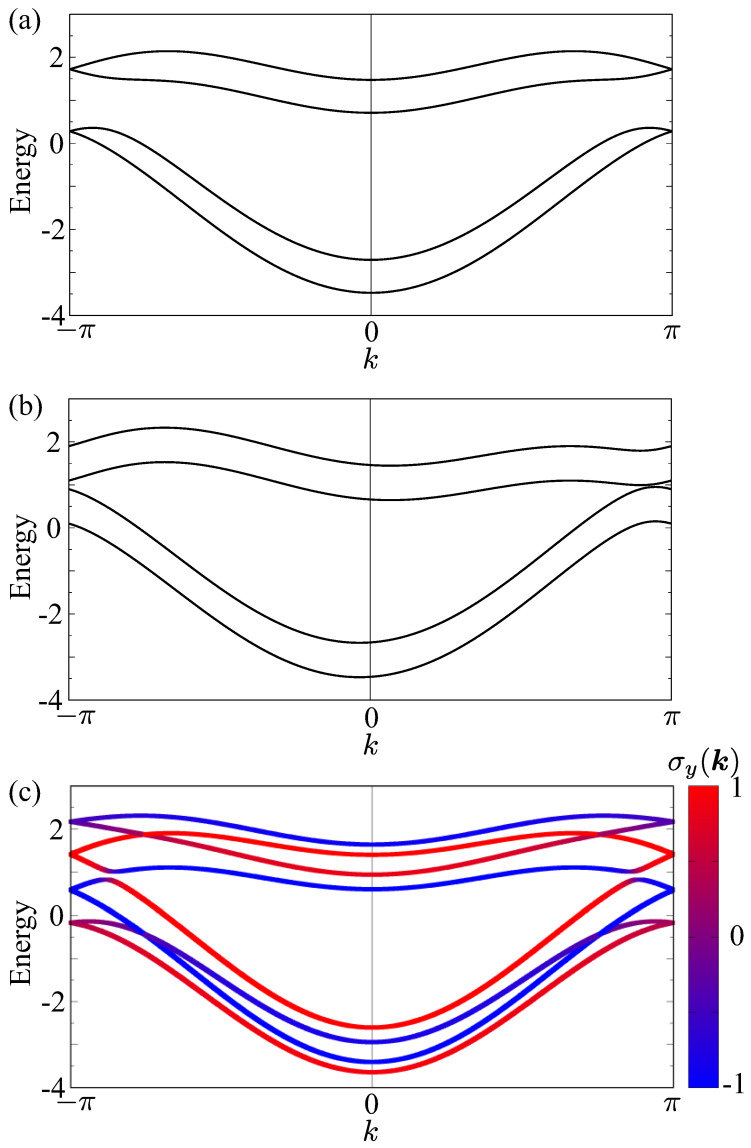
Electronic band structures in (**a**) the paramagnetic state at $\alpha_{1}=0.3$, $\alpha_{2}=0.15$, and $h^{\mathrm{FMT}}=h^{\mathrm{AFMT}}=0$, (**b**) the ferromagnetic toroidal dipole (uniform MTD) state at $\alpha_{1}=0.3$, $\alpha_{2}=0$, $h^{\mathrm{FMT}}=0.5$, and $h^{\mathrm{AFMT}}=0$, and (**c**) antiferromagnetic toroidal dipole (staggered MTD) state at $\alpha_{1}=0$, $\alpha_{2}=0.15$, $h^{\mathrm{FMT}}=0$, and $h^{\mathrm{AFMT}}=0.5$. In (**c**), the color shows the momentum-resolved *y*-spin polarization.

We show the electronic band structure obtained by diagonalizing the Hamiltonian in Equation (2). We set $t_{1}=1$, $t_{2}=0.5$, and $t_{z}=0.4$ without loss of generality; the characteristic points in the following band structures are not affected by the different choices of hopping parameters. Figure 4a shows the band structure in the paramagnetic state at $\alpha_{1}=0.3$, $\alpha_{2}=0.15$, and $h^{\mathrm{FMT}}=h^{\mathrm{AFMT}}=0$. There are four bands, each of which is doubly degenerated owing to the presence of the PT symmetry; *P* and *T* stand for the spatial inversion and time-reversal operations, respectively.

For the uniform MTD ordering with $h^{\mathrm{FMT}}=0.5$ and $h^{\mathrm{AFMT}}=0$, the band structure at $\alpha_{1}=0.3$ and $\alpha_{2}=0$ is shown in Figure 4b. Similarly to the band structure in Figure 4a, each band is degenerated with keeping the PT symmetry. Meanwhile, the band dispersion is asymmetric with respect to k=0, which means that *k* and −k are not equivalent to each other. This is because of the breaking of both spatial inversion and time-reversal symmetries under the MTD. Reflecting the asymmetric band modulation, the system exhibits the nonreciprocal transport along the *x* direction [27,81].

Figure 4c shows the band structure under the staggered MTD ordering with $h^{\mathrm{AFMT}}=0.5$ and $h^{\mathrm{FMT}}=0$ at $\alpha_{1}=0$ and $\alpha_{2}=0.15$. In contrast to the band structures in Figure 4a,b, the spin degeneracy in the band is lifted; the *y*-spin polarization occurs. This result indicates that the mean field to induce the staggered MTD ordering gives rise to spontaneous magnetization along the direction perpendicular to both the spin and MTD moments, which is consistent with the symmetry argument in Section 2.1.

The above results show that the uniform and staggered MTD orderings lead to asymmetric band deformation and spin splitting, respectively. On the other hand, the necessary model parameters to induce such band modulations are different from each other. In the case of the uniform MTD ordering, the ASOI $\alpha_{1}$ plays an important role, while $\alpha_{2}$ is not necessary for inducing the asymmetric band modulation. The opposite tendency appears in the staggered MTD ordering; $\alpha_{2}$ is the essential model parameter to cause the spin splitting instead of $\alpha_{1}$. In other words, the ASOI, whose spin component is parallel to the MTD direction, leads to a significant contribution to causing the band modulations in both cases. In particular, a larger ASOI between the inter-zigzag chain is important in order to obtain the large magnetization under the staggered MTD ordering.

### 3.2. Spontaneous Magnetization

We investigate the behavior of the uniform magnetization under the staggered MTD ordering when the model parameters are varied. In this section, we fix $\alpha_{1}=0$, $\alpha_{2}=0.15$, and $h^{\mathrm{FMT}}=0$, and change $h^{\mathrm{AFMT}}$ and the electron filling per site $n_{e}$; $n_{e}=2$ corresponds to full filling.

**Figure 5:**
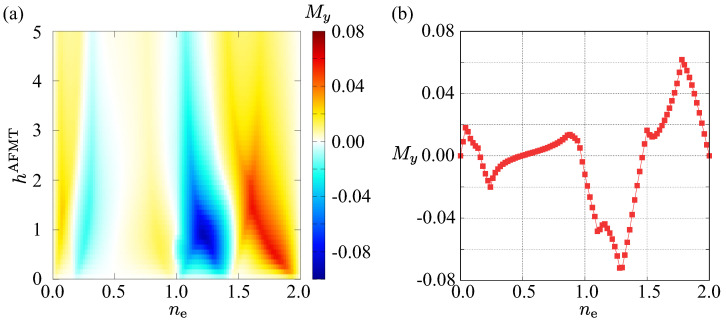
(**a**) Filling $n_{e}$ and the mean field $h^{\mathrm{AFMT}}$ dependence of the uniform magnetization along the *y* direction $M_{y}$. The model parameters are the same as those used in Figure 4c. (**b**) $n_{e}$ dependence of $M_{y}$ at $h^{\mathrm{AFMT}}=0.5$.

Figure 5a shows the uniform magnetzation along the *y* direction, $M_{y}$, in the plane of $n_{e}$ and $h^{\mathrm{AFMT}}$. In almost all of the regions, $M_{y}$ becomes nonzero, indicating that the staggered MTD ordering induces uniform magnetization. Although the $n_{e}$ dependence is complicated, as shown in the case of $h^{\mathrm{AFMT}}=0.5$ in Figure 5b, one finds that the $h^{\mathrm{AFMT}}$ dependence is small.

Finally, let us comment on physical phenomena under the staggered MTD ordering. Since spontaneous magnetization appears once the staggered MTD ordering occurs, one can expect ferromagnetic-related physical phenomena, such as the anomalous Hall/Nernst effect and magneto-optical Kerr effect, even in AFM structures [82] when the AFM moment is effectively couplied to the ASOI, which have been recently found in various materials like La*M*O_3_ (M= Cr, Mn, and Fe) [83], Mn_3_Ir [84,85,86], Mn_3_Sn [87,88,89,90,91,92], antiperovskite Mn_3_*A*N (A= Ga, Sn, and Ni) [93,94,95,96,97], NdMnP [98,99], the pyrochlore oxides [100,101], the bilayer MnPSe_3_ [102], κ-type organic conductors [103], Ce_2_CuGe_6_ [104], and other systems [105,106,107,108,109,110,111,112,113,114,115]. The staggered MTD system provides another promising system to induce such physical phenomena in AFMs.

## 4. Conclusions

To summarize, we have investigated physical properties in AFMs from the multipole point of view. We have clarified that the staggered alignment of the MTD accompanies spontaneous magnetization perpendicular to the MTD moment based on symmetry analysis and microscopic model calculations. By considering the minimal model on the bilayer zigzag chain, we have shown that spontaneous magnetization is induced under the staggered MTD ordering when the effect of the spin-orbit coupling along the MTD moment direction is taken into account. The induced magnetization indicates emergent ferromagnetic-related physical phenomena like the anomalous Hall effect, which will be useful for future exploration of functional AFM materials based on the MTD moment.

## Figures and Tables

**Table 1:** Correspondence between the irreducible representation (Irrep.) and multipoles under the zigzag-chain structure belonging to the point group *D*_2h_. The staggered AFM structure on the zigzag chain leads to the ferroic alignment of multipoles; *T*_0_ (*M*_0_) stands for the magnetic toroidal (magnetic) monopole, while $\left(T_{x},\;T_{y},\;T_{z}\right)$ [$\left(M_{x},\;M_{y},\;M_{z}\right)$] stands for the magnetic toroidal (magnetic) dipole. The superscript of the Irrep. denotes the time-reversal parity. In the columns “FM stacking” and “AFM stacking”, *x*, *y*, and *z* mean the direction of the AFM moment, where the staggered AFM structure on the zigzag chain is stacked ferromagnetically and antiferromagnetically, respectively. “FMT” and “AFMT” in the rightmost column correspond to the ferromagnetic toroidal dipole and antiferromagnetic toroidal dipole orderings, respectively, which are analyzed in this study. It is noted that the AFM ordering on the single zigzag chain gives the same result as the FM stacking case.

Irrep.	Multipole	FM Stacking	AFM Stacking	Note
$A_{g}^{-}$	$T_{0}$	−	*x*	
$B_{1g}^{-}$	$M_{z}$	−	*y*	
$B_{2g}^{-}$	$M_{y}$	−	*z*	AFMT
$B_{3g}^{-}$	$M_{x}$	−	−	
$A_{u}^{-}$	$M_{0}$	*y*	−	
$B_{1u}^{-}$	$T_{z}$	*x*	−	
$B_{2u}^{-}$	$T_{y}$	−	−	
$B_{3u}^{-}$	$T_{x}$	*z*	−	FMT

## Data Availability

The original contributions presented in the study are included in the article, further inquiries can be directed to the corresponding author.

## References

[1] Dubovik V., Cheshkov A. (1975). Multipole expansion in classical and quantum field theory and radiation. Sov. J. Part. Nucl..

[2] Dubovik V., Tugushev V. (1990). Toroid moments in electrodynamics and solid-state physics. Phys. Rep..

[3] Gorbatsevich A., Kopaev Y.V. (1994). Toroidal order in crystals. Ferroelectrics.

[4] Kopaev Y.V. (2009). Toroidal ordering in crystals. Physics-Uspekhi.

[5] Spaldin N.A., Fiebig M., Mostovoy M. (2008). The toroidal moment in condensed-matter physics and its relation to the magnetoelectric effect. J. Phys. Condens. Matter.

[6] Papasimakis N., Fedotov V., Savinov V., Raybould T., Zheludev N. (2016). Electromagnetic toroidal excitations in matter and free space. Nat. Mater..

[7] Talebi N., Guo S., van Aken P.A. (2018). Theory and applications of toroidal moments in electrodynamics: Their emergence, characteristics, and technological relevance. Nanophotonics.

[8] Cheong S.W., Talbayev D., Kiryukhin V., Saxena A. (2018). Broken symmetries, non-reciprocity, and multiferroicity. npj Quantum Mater..

[9] Kusunose H., Hayami S. (2022). Generalization of microscopic multipoles and cross-correlated phenomena by their orderings. J. Phys. Condens. Matter.

[10] Hayami S., Kusunose H. (2024). Unified description of electronic orderings and cross correlations by complete multipole representation. J. Phys. Soc. Jpn..

[11] Xu X., Huang F.T., Cheong S.W. (2024). Magnetic toroidicity. J. Phys. Condens. Matter.

[12] Azimi-Mousolou V., Bergman A., Delin A., Eriksson O., Pereiro M., Thonig D., Sjöqvist E. (2024). Quantum spin systems: Toroidal classification and geometric duality. Phys. Rev. B.

[13] Popov Y.F., Kadomtseva A., Belov D., Vorob’ev G., Zvezdin A. (1999). Magnetic-field-induced toroidal moment in the magnetoelectric Cr2O3. J. Exp. Theor. Phys. Lett..

[14] Schmid H. (2001). On ferrotoroidics and electrotoroidic, magnetotoroidic and piezotoroidic effects*. Ferroelectrics.

[15] Ederer C., Spaldin N.A. (2007). Towards a microscopic theory of toroidal moments in bulk periodic crystals. Phys. Rev. B.

[16] Yanase Y. (2014). Magneto-Electric Effect in Three-Dimensional Coupled Zigzag Chains. J. Phys. Soc. Jpn..

[17] Thöle F., Spaldin N.A. (2018). Magnetoelectric multipoles in metals. Philos. Trans. R. Soc. A.

[18] Miyahara S., Furukawa N. (2012). Nonreciprocal Directional Dichroism and Toroidalmagnons in Helical Magnets. J. Phys. Soc. Jpn..

[19] Miyahara S., Furukawa N. (2014). Theory of magneto-optical effects in helical multiferroic materials via toroidal magnon excitation. Phys. Rev. B.

[20] Hayami S., Kusunose H., Motome Y. (2016). Asymmetric Magnon Excitation by Spontaneous Toroidal Ordering. J. Phys. Soc. Jpn..

[21] Matsumoto T., Hayami S. (2020). Nonreciprocal magnons due to symmetric anisotropic exchange interaction in honeycomb antiferromagnets. Phys. Rev. B.

[22] Sawada K., Nagaosa N. (2005). Optical Magnetoelectric Effect in Multiferroic Materials: Evidence for a Lorentz Force Acting on a Ray of Light. Phys. Rev. Lett..

[23] Kawaguchi H., Tatara G. (2016). Effective Hamiltonian theory for nonreciprocal light propagation in magnetic Rashba conductor. Phys. Rev. B.

[24] Watanabe H., Yanase Y. (2020). Nonlinear electric transport in odd-parity magnetic multipole systems: Application to Mn-based compounds. Phys. Rev. Res..

[25] Watanabe H., Yanase Y. (2021). Photocurrent response in parity-time symmetric current-ordered states. Phys. Rev. B.

[26] Suzuki Y. (2022). Tunneling spin current in systems with spin degeneracy. Phys. Rev. B.

[27] Yatsushiro M., Oiwa R., Kusunose H., Hayami S. (2022). Analysis of model-parameter dependences on the second-order nonlinear conductivity in *PT*-symmetric collinear antiferromagnetic metals with magnetic toroidal moment on zigzag chains. Phys. Rev. B.

[28] Kondo H., Akagi Y. (2022). Nonlinear magnon spin Nernst effect in antiferromagnets and strain-tunable pure spin current. Phys. Rev. Res..

[29] Hayami S., Yatsushiro M., Kusunose H. (2022). Nonlinear spin Hall effect in *PT*-symmetric collinear magnets. Phys. Rev. B.

[30] Mentink S.A.M., Drost A., Nieuwenhuys G.J., Frikkee E., Menovsky A.A., Mydosh J.A. (1994). Magnetic Ordering and Frustration in Hexagonal UNi_4_B. Phys. Rev. Lett..

[31] Oyamada A., Kondo M., Fukuoka K., Itou T., Maegawa S., Li D.X., Haga Y. (2007). NMR studies of the partially disordered state in a triangular antiferromagnet UNi_4_B. J. Phys. Condens. Matter.

[32] Yanagisawa T., Matsumori H., Saito H., Hidaka H., Amitsuka H., Nakamura S., Awaji S., Gorbunov D.I., Zherlitsyn S., Wosnitza J. (2021). Electric Quadrupolar Contributions in the Magnetic Phases of *UNi*_4_B. Phys. Rev. Lett..

[33] Ishitobi T., Hattori K. (2023). Triple-*Q* partial magnetic orders induced by quadrupolar interactions: Triforce order scenario for UNi_4_B. Phys. Rev. B.

[34] Saito H., Uenishi K., Miura N., Tabata C., Hidaka H., Yanagisawa T., Amitsuka H. (2018). Evidence of a New Current-Induced Magnetoelectric Effect in a Toroidal Magnetic Ordered State of UNi_4_B. J. Phys. Soc. Jpn..

[35] Ota K., Shimozawa M., Muroya T., Miyamoto T., Hosoi S., Nakamura A., Homma Y., Honda F., Aoki D., Izawa K. (2022). Zero-field current-induced Hall effect in ferrotoroidic metal. arXiv.

[36] Folen V.J., Rado G.T., Stalder E.W. (1961). Anisotropy of the Magnetoelectric Effect in Cr_2_O_3_. Phys. Rev. Lett..

[37] Krotov S., Kadomtseva A., Popov Y.F., Zvezdin A., Vorob’ev G., Belov D. (2001). Magnetoelectric interactions and induced toroidal ordering in Cr_2_O_3_. J. Magn. Magn. Mater..

[38] Arima T., Jung J.H., Matsubara M., Kubota M., He J.P., Kaneko Y., Tokura Y. (2005). Resonant magnetoelectric X-ray scattering in GaFeO_3_: Observation of ordering of toroidal moments. J. Phys. Soc. Jpn..

[39] Staub U., Bodenthin Y., Piamonteze C., García-Fernández M., Scagnoli V., Garganourakis M., Koohpayeh S., Fort D., Lovesey S.W. (2009). Parity- and time-odd atomic multipoles in magnetoelectric GaFeO_3_ as seen via soft x-ray Bragg diffraction. Phys. Rev. B.

[40] Staub U., Piamonteze C., Garganourakis M., Collins S.P., Koohpayeh S.M., Fort D., Lovesey S.W. (2012). Ferromagnetic-type order of atomic multipoles in the polar ferrimagnetic GaFeO_3_. Phys. Rev. B.

[41] Nie Y.m. (2016). First-principles approach to investigate toroidal property of magnetoelectric multiferroic GaFeO_3_. J. Appl. Phys..

[42] Van Aken B.B., Rivera J.P., Schmid H., Fiebig M. (2007). Observation of ferrotoroidic domains. Nature.

[43] Zimmermann A.S., Meier D., Fiebig M. (2014). Ferroic nature of magnetic toroidal order. Nat. Commun..

[44] Fogh E., Zaharko O., Schefer J., Niedermayer C., Holm-Dahlin S., Sørensen M.K., Kristensen A.B., Andersen N.H., Vaknin D., Christensen N.B. (2019). Dzyaloshinskii-Moriya interaction and the magnetic ground state in magnetoelectric LiCoPO_4_. Phys. Rev. B.

[45] Tóth B., Kocsis V., Tokunaga Y., Taguchi Y., Tokura Y., Bordács S. (2024). Imaging antiferromagnetic domains in LiCoPO_4_ via the optical magnetoelectric effect. Phys. Rev. B.

[46] Murakawa H., Onose Y., Miyahara S., Furukawa N., Tokura Y. (2010). Ferroelectricity Induced by Spin-Dependent Metal-Ligand Hybridization in Ba_2_CoGe_2_O_7_. Phys. Rev. Lett..

[47] Yamauchi K., Barone P., Picozzi S. (2011). Theoretical investigation of magnetoelectric effects in Ba_2_CoGe_2_O_7_. Phys. Rev. B.

[48] Toledano P., Khalyavin D.D., Chapon L.C. (2011). Spontaneous toroidal moment and field-induced magnetotoroidic effects in Ba_2_CoGe_2_O_7_. Phys. Rev. B.

[49] Hutanu V., Sazonov A., Murakawa H., Tokura Y., Náfrádi B., Chernyshov D. (2011). Symmetry and structure of multiferroic Ba_2_CoGe_2_O_7_. Phys. Rev. B.

[50] Baum M., Schmalzl K., Steffens P., Hiess A., Regnault L.P., Meven M., Becker P., Bohatý L., Braden M. (2013). Controlling toroidal moments by crossed electric and magnetic fields. Phys. Rev. B.

[51] Tolédano P., Ackermann M., Bohatý L., Becker P., Lorenz T., Leo N., Fiebig M. (2015). Primary ferrotoroidicity in antiferromagnets. Phys. Rev. B.

[52] Lee C., Kang J., Hong J., Shim J.H., Whangbo M.H. (2014). Analysis of the difference between the pyroxenes LiFeSi_2_O_6_ and LiFeGe_2_O_6_ in their spin order, spin orientation, and ferrotoroidal order. Chem. Mater..

[53] Wadley P., Novák V., Campion R., Rinaldi C., Martí X., Reichlová H., Železnỳ J., Gazquez J., Roldan M., Varela M. (2013). Tetragonal phase of epitaxial room-temperature antiferromagnet CuMnAs. Nat. Commun..

[54] Wadley P., Howells B., Železnỳ J., Andrews C., Hills V., Campion R.P., Novák V., Olejník K., Maccherozzi F., Dhesi S. (2016). Electrical switching of an antiferromagnet. Science.

[55] Godinho J., Reichlová H., Kriegner D., Novák V., Olejník K., Kašpar Z., Šobáň Z., Wadley P., Campion R., Otxoa R. (2018). Electrically induced and detected Néel vector reversal in a collinear antiferromagnet. Nat. Commun..

[56] Manchon A., Železný J., Miron I.M., Jungwirth T., Sinova J., Thiaville A., Garello K., Gambardella P. (2019). Current-induced spin-orbit torques in ferromagnetic and antiferromagnetic systems. Rev. Mod. Phys..

[57] Wang C., Gao Y., Xiao D. (2021). Intrinsic Nonlinear Hall Effect in Antiferromagnetic Tetragonal CuMnAs. Phys. Rev. Lett..

[58] Ding L., Xu X., Jeschke H.O., Bai X., Feng E., Alemayehu A.S., Kim J., Huang F.T., Zhang Q., Ding X. (2021). Field-tunable toroidal moment in a chiral-lattice magnet. Nat. Commun..

[59] Xu X., Huang F.T., Admasu A.S., Kratochvílová M., Chu M.W., Park J.G., Cheong S.W. (2022). Multiple ferroic orders and toroidal magnetoelectricity in the chiral magnet BaCoSiO_4_. Phys. Rev. B.

[60] Nakamura R., Aoki I., Kimura K. (2024). Ferrotoroidic State Induced by Structural Rotation and Falsely Chiral Antiferromagnetism in PbMn_2_Ni_6_Te_3_O_18_. J. Phys. Soc. Jpn..

[61] Hayami S., Kusunose H., Motome Y. (2015). Spontaneous Multipole Ordering by Local Parity Mixing. J. Phys. Soc. Jpn..

[62] Cysne T.P., Guimarães F.S.M., Canonico L.M., Rappoport T.G., Muniz R.B. (2021). Orbital magnetoelectric effect in zigzag nanoribbons of *p*-band systems. Phys. Rev. B.

[63] Li X., Cao T., Niu Q., Shi J., Feng J. (2013). Coupling the valley degree of freedom to antiferromagnetic order. Proc. Natl. Acad. Sci. USA.

[64] Hayami S., Kusunose H., Motome Y. (2014). Spontaneous parity breaking in spin-orbital coupled systems. Phys. Rev. B.

[65] Hayami S., Kusunose H., Motome Y. (2016). Emergent spin-valley-orbital physics by spontaneous parity breaking. J. Phys. Condens. Matter.

[66] Yanagi Y., Kusunose H. (2017). Optical Selection Rules in Spin–Orbit Coupled Systems on Honeycomb Lattice. J. Phys. Soc. Jpn..

[67] Yanagi Y., Hayami S., Kusunose H. (2018). Manipulating the magnetoelectric effect: Essence learned from Co_4_Nb_2_O_9_. Phys. Rev. B.

[68] Oishi R., Umeo K., Shimura Y., Onimaru T., Strydom A.M., Takabatake T. (2021). Antiferromagnetic order in the honeycomb Kondo lattice CePt_6_Al_3_ induced by Pd substitution. Phys. Rev. B.

[69] Hayami S., Kusunose H., Motome Y. (2018). Emergent odd-parity multipoles and magnetoelectric effects on a diamond structure: Implication for the 5*d* transition metal oxides *A* OsO_4_ (*A* = K, Rb, and Cs). Phys. Rev. B.

[70] Ishitobi T., Hattori K. (2019). Magnetoelectric Effects and Charge-Imbalanced Solenoids: Antiferro Quadrupole Orders in a Diamond Structure. J. Phys. Soc. Jpn..

[71] Yamaura J.i., Hiroi Z. (2019). Crystal structure and magnetic properties of the 5*d* transition metal oxides *A* OsO_4_ (*A* = K, Rb, Cs). Phys. Rev. B.

[72] Paramekanti A., Maharaj D.D., Gaulin B.D. (2020). Octupolar order in *d*-orbital Mott insulators. Phys. Rev. B.

[73] Maharaj D.D., Sala G., Stone M.B., Kermarrec E., Ritter C., Fauth F., Marjerrison C.A., Greedan J.E., Paramekanti A., Gaulin B.D. (2020). Octupolar versus Néel Order in Cubic 5*d*^2^ Double Perovskites. Phys. Rev. Lett..

[74] Winkler R., Zülicke U. (2023). Theory of electric, magnetic, and toroidal polarizations in crystalline solids with applications to hexagonal lonsdaleite and cubic diamond. Phys. Rev. B.

[75] Hitomi T., Yanase Y. (2014). Electric Octupole Order in Bilayer Ruthenate Sr3Ru2O7. J. Phys. Soc. Jpn..

[76] Hitomi T., Yanase Y. (2016). Electric octupole order in bilayer Rashba system. J. Phys. Soc. Jpn..

[77] Kirikoshi A., Hayami S. (2023). Microscopic mechanism for intrinsic nonlinear anomalous Hall conductivity in noncollinear antiferromagnetic metals. Phys. Rev. B.

[78] Yatsushiro M., Kusunose H., Hayami S. (2021). Multipole classification in 122 magnetic point groups for unified understanding of multiferroic responses and transport phenomena. Phys. Rev. B.

[79] Kusunose H., Oiwa R., Hayami S. (2023). Symmetry-adapted modeling for molecules and crystals. Phys. Rev. B.

[80] Hayami S., Kusunose H. (2023). Time-reversal switching responses in antiferromagnets. Phys. Rev. B.

[81] Tokura Y., Nagaosa N. (2018). Nonreciprocal responses from non-centrosymmetric quantum materials. Nat. Commun..

[82] Baltz V., Manchon A., Tsoi M., Moriyama T., Ono T., Tserkovnyak Y. (2018). Antiferromagnetic spintronics. Rev. Mod. Phys..

[83] Solovyev I.V. (1997). Magneto-optical effect in the weak ferromagnets LaMO_3_ (M= Cr, Mn, and Fe). Phys. Rev. B.

[84] Chen H., Niu Q., MacDonald A.H. (2014). Anomalous Hall Effect Arising from Noncollinear Antiferromagnetism. Phys. Rev. Lett..

[85] Chen H., Wang T.C., Xiao D., Guo G.Y., Niu Q., MacDonald A.H. (2020). Manipulating anomalous Hall antiferromagnets with magnetic fields. Phys. Rev. B.

[86] Kurita K., Koretsune T. (2024). X-ray Magnetic Circular Dichroism Arising from the Magnetic Dipole Moment in Antiferromagnets. J. Phys. Soc. Jpn..

[87] Nakatsuji S., Kiyohara N., Higo T. (2015). Large anomalous Hall effect in a non-collinear antiferromagnet at room temperature. Nature.

[88] Suzuki M.T., Koretsune T., Ochi M., Arita R. (2017). Cluster multipole theory for anomalous Hall effect in antiferromagnets. Phys. Rev. B.

[89] Ikhlas M., Tomita T., Koretsune T., Suzuki M.T., Nishio-Hamane D., Arita R., Otani Y., Nakatsuji S. (2017). Large anomalous Nernst effect at room temperature in a chiral antiferromagnet. Nat. Phys..

[90] Kuroda K., Tomita T., Suzuki M.T., Bareille C., Nugroho A., Goswami P., Ochi M., Ikhlas M., Nakayama M., Akebi S. (2017). Evidence for magnetic Weyl fermions in a correlated metal. Nat. Mater..

[91] Higo T., Man H., Gopman D.B., Wu L., Koretsune T., van’t Erve O.M., Kabanov Y.P., Rees D., Li Y., Suzuki M.T. (2018). Large magneto-optical Kerr effect and imaging of magnetic octupole domains in an antiferromagnetic metal. Nat. Photonics.

[92] Kimata M., Sasabe N., Kurita K., Yamasaki Y., Tabata C., Yokoyama Y., Kotani Y., Ikhlas M., Tomita T., Amemiya K. (2021). X-ray study of ferroic octupole order producing anomalous Hall effect. Nat. Commun..

[93] Gurung G., Shao D.F., Paudel T.R., Tsymbal E.Y. (2019). Anomalous Hall conductivity of noncollinear magnetic antiperovskites. Phys. Rev. Mater..

[94] Zhou X., Hanke J.P., Feng W., Li F., Guo G.Y., Yao Y., Blügel S., Mokrousov Y. (2019). Spin-order dependent anomalous Hall effect and magneto-optical effect in the noncollinear antiferromagnets Mn_3_*X*N with *X* = Ga, Zn, Ag, or Ni. Phys. Rev. B.

[95] Boldrin D., Samathrakis I., Zemen J., Mihai A., Zou B., Johnson F., Esser B.D., McComb D.W., Petrov P.K., Zhang H. (2019). Anomalous Hall effect in noncollinear antiferromagnetic Mn_3_NiN thin films. Phys. Rev. Mater..

[96] Huyen V.T.N., Suzuki M.T., Yamauchi K., Oguchi T. (2019). Topology analysis for anomalous Hall effect in the noncollinear antiferromagnetic states of Mn_3_⁢ *A⁢*N (*A⁢* = Ni, Cu, Zn, Ga, Ge, Pd, In, Sn, Ir, Pt). Phys. Rev. B.

[97] You Y., Bai H., Feng X., Fan X., Han L., Zhou X., Zhou Y., Zhang R., Chen T., Pan F. (2021). Cluster magnetic octupole induced out-of-plane spin polarization in antiperovskite antiferromagnet. Nat. Commun..

[98] Kotegawa H., Kuwata Y., Huyen V.T.N., Arai Y., Tou H., Matsuda M., Takeda K., Sugawara H., Suzuki M.T. (2023). Large anomalous Hall effect and unusual domain switching in an orthorhombic antiferromagnetic material NbMnP. npj Quantum Mater..

[99] Arai Y., Hayashi J., Takeda K., Tou H., Sugawara H., Kotegawa H. (2024). Intrinsic Anomalous Hall Effect Arising from Antiferromagnetism as Revealed by High-Quality NbMnP. J. Phys. Soc. Jpn..

[100] Tomizawa T., Kontani H. (2009). Anomalous Hall effect in the *t*_2g_ orbital kagome lattice due to noncollinearity: Significance of the orbital Aharonov-Bohm effect. Phys. Rev. B.

[101] Kim W.J., Oh T., Song J., Ko E.K., Li Y., Mun J., Kim B., Son J., Yang Z., Kohama Y. (2020). Strain engineering of the magnetic multipole moments and anomalous Hall effect in pyrochlore iridate thin films. Sci. Adv..

[102] Sivadas N., Okamoto S., Xiao D. (2016). Gate-Controllable Magneto-optic Kerr Effect in Layered Collinear Antiferromagnets. Phys. Rev. Lett..

[103] Naka M., Hayami S., Kusunose H., Yanagi Y., Motome Y., Seo H. (2020). Anomalous Hall effect in *κ*-type organic antiferromagnets. Phys. Rev. B.

[104] Kotegawa H., Tanaka H., Takeuchi Y., Tou H., Sugawara H., Hayashi J., Takeda K. (2024). Large Anomalous Hall Conductivity Derived from an *f*-Electron Collinear Antiferromagnetic Structure. Phys. Rev. Lett..

[105] Šmejkal L., González-Hernández R., Jungwirth T., Sinova J. (2020). Crystal time-reversal symmetry breaking and spontaneous Hall effect in collinear antiferromagnets. Sci. Adv..

[106] Yamasaki Y., Nakao H., Arima T.h. (2020). Augmented Magnetic Octupole in Kagomé 120-degree Antiferromagnets Detectable via X-ray Magnetic Circular Dichroism. J. Phys. Soc. Jpn..

[107] Akiba K., Iwamoto K., Sato T., Araki S., Kobayashi T.C. (2020). Anomalous Hall effect triggered by pressure-induced magnetic phase transition in *α*-Mn. Phys. Rev. Res..

[108] Hayami S., Kusunose H. (2021). Essential role of the anisotropic magnetic dipole in the anomalous Hall effect. Phys. Rev. B.

[109] Chen H. (2022). Electronic chiralization as an indicator of the anomalous Hall effect in unconventional magnetic systems. Phys. Rev. B.

[110] Lei C., Trevisan T.V., Heinonen O., McQueeney R.J., MacDonald A.H. (2022). Quantum anomalous Hall effect in perfectly compensated collinear antiferromagnetic thin films. Phys. Rev. B.

[111] Sasabe N., Kimata M., Nakamura T. (2021). Presence of X-Ray Magnetic Circular Dichroism Signal for Zero-Magnetization Antiferromagnetic State. Phys. Rev. Lett..

[112] Gonzalez Betancourt R.D., Zubáč J., Gonzalez-Hernandez R., Geishendorf K., Šobáň Z., Springholz G., Olejník K., Šmejkal L., Sinova J., Jungwirth T. (2023). Spontaneous Anomalous Hall Effect Arising from an Unconventional Compensated Magnetic Phase in a Semiconductor. Phys. Rev. Lett..

[113] Sasabe N., Mizumaki M., Uozumi T., Yamasaki Y. (2023). Ferroic Order for Anisotropic Magnetic Dipole Term in Collinear Antiferromagnets of (*t*_2g_)^4^ System. Phys. Rev. Lett..

[114] Attias L., Levchenko A., Khodas M. (2024). Intrinsic anomalous Hall effect in altermagnets. Phys. Rev. B.

[115] Hariki A., Dal Din A., Amin O.J., Yamaguchi T., Badura A., Kriegner D., Edmonds K.W., Campion R.P., Wadley P., Backes D. (2024). X-Ray Magnetic Circular Dichroism in Altermagnetic *α*-MnTe. Phys. Rev. Lett..

